# Postaxial hypoplasia of the lower extremity associated with congenital dislocation of the patella: A case report

**DOI:** 10.1097/MD.0000000000029283

**Published:** 2022-06-24

**Authors:** Sergio Charles-Lozoya, Gibran Ruíz-Zenteno, Héctor Cobos-Aguilar, María Lizcano-Martínez, Edgar Manilla-Muñoz, Miguel Leonardo De La Parra-Márquez, Adrián García-Hernández

**Affiliations:** a Health Science Division, Division of Plastic and Reconstructive Surgery, Unit of Hip and Pelvis Orthopedic Surgery, Hospital de Traumatología y Ortopedia No. 21, Instituto Mexicano del Seguro Social (IMSS), Monterrey, N.L., México; b Health Science Division, Vice-Rectory of Health Sciences, Universidad de Monterrey, San Pedro Garza García, N.L., México.

**Keywords:** congenital dislocation of the patella, fibular hemimelia, postaxial hypoplasia, surgery

## Abstract

**Rationale:**

Evaluation of clinical and radiologic abnormalities in patients with postaxial hypoplasia of the lower extremity (PHLE) for treatment decisions represents a major challenge, which is more complicated when PHLE is associated with congenital dislocation of the patella.

**Patient concerns:**

: Herein, we present the case of an 8-year-old female patient with evident length inequality in her left lower extremity and inability to walk.

**Diagnoses:**

Radiological evaluation revealed PHLE with fibular hemimelia, proximal femoral focal deficiency, tarsal coalition, and congenital patellar dislocation of the patella. The right lower extremity was also affected by fibular hemimelia.

**Interventions and outcomes:**

Surgical management included the Roux-Goldthwait technique for patellofemoral joint realignment, a medial knee stapled with Blount technique, and femur enlargement using the Wagner technique. The results from surgical intervention included a left femoral elongation of 6.7 cm featuring callus with angulation, displacement, and a discrepancy of 5 cm between femurs with a flexor contraction in the knee of −15° and a centralized knee.

**Lesson:**

PHLE accompanied by congenital dislocation of the patella has not been extensively described in the literature; therefore, there is no established management. Starting reconstruction at an early age, together with an adequate classification of the deformity, are essential factors when opting for limb reconstruction.

## 1 Introduction

Postaxial hypoplasia of the lower extremity (PHLE) is part of a group of diseases of unclear etiology, with an incidence of 5.7 to 20 cases per 1 million births, including fibular hemimelia (FH), proximal femoral focal deficiency (PFFD), and tarsal coalition (TC).^[1,2]^ When possible, surgical treatment should be reconstructive and focused on restoring normal limb alignment and length with a stable plantigrade foot for effective gait.^[3]^

Congenital dislocation of the patella (CDP) is rare and its association with PHLE is even greater. CDP is characterized by stiffness, short quadriceps, femoropatellar dysplasia, flexion contracture, genu valgum, external tibial torsion, and foot deformities. CDP may occur as an isolated clinical entity or is associated with polymalformative syndrome.^[4,5]^

Under both conditions, an effective reconstructive plan can only be obtained using a combination of procedures. As an experienced multidisciplinary team, we discuss the key aspects that must be considered when attempting to reconstruct a severely deformed extremity.

## 2 Case presentation

We present the case of an 8-year-old girl with a history of PHLE, including FH, PFFD, and TC, complicated with CDP. A product of the mother's first pregnancy with no prenatal control, she was born at 38 weeks of gestation by cesarean section due to severe oligohydramnios, without family history of skeletal anomalies. The following conditions were diagnosed at birth: amputation of the right forearm by amniotic bands, bilateral FH, agenesis of the 4th and 5th rays in the left foot and the 5th ray in the right foot, congenital left clubfoot, and PFFD. She was treated using the Ponseti method and subcutaneous heel cord tenotomy for congenital talipes equinovarus.

On physical examination, the patient was in a wheelchair, with an evident leg length inequality of approximately 9.7 cm on her left lower extremity, left knee with deviation in valgus, a flexion contracture in the range of −20°, with relative stiffness, and stable knee with an anterior cruciate ligament absent. In addition, irreducible lateral dislocation of the left patella was observed. Knee range of motion during passive movements was 120° flexion and −20° extension without pain. Anteromedial bowing with a length discrepancy was observed in the left leg.

Her left ankle and hindfoot were lateral to the proximal malleolus in the ankle mortise, and slight valgus (10°) was present with associated instability and hypermobility. Her foot was hypotrophic, shortened, and slimmed down, with agenesis of the 4th and 5th toes, and a full range of motion at the first metatarsophalangeal joint.

**Figure 1:**
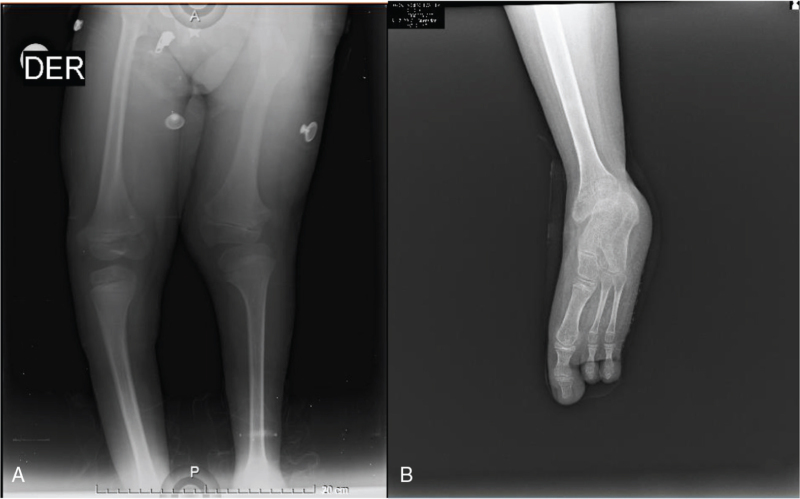
(A) Anteroposterior femur, tibia X ray, bilateral fibular hemimelia with left short femur. (B) Oblique left foot radiograph demonstrates deficiency of the fourth and fifth ray, only 1 cuneiform can be observed and tarsal coalition.

Radiographic examination showed an absence of the 4th and 5th rays, as well as failure in the formation of the cuboid, lateral, and medial cuneiform bones, with calcaneus, talus, and medial cuneiform bones present in the TC. The articular surface of the distal tibia and the surface of the fused talocalcaneal bone were flat. The distal tibial epiphysis was wedge shaped and narrower on the lateral side. The fibula was completely absent in both legs with FH, and the deformities were classified as type II under the Achterman and Kalamchi Classification,^[6]^ discreet anteromedial bowing in right tibia was observed. Limb length discrepancy was caused mainly by the short femur; the tibia did not contribute as much to the shortening (Fig. 1). The patella was lateral, and the knee in valgus deviation and femoral congenital limb deficiency were classified as Pappas VIII.^[7]^

## 3 Surgical treatment

**Figure 2:**
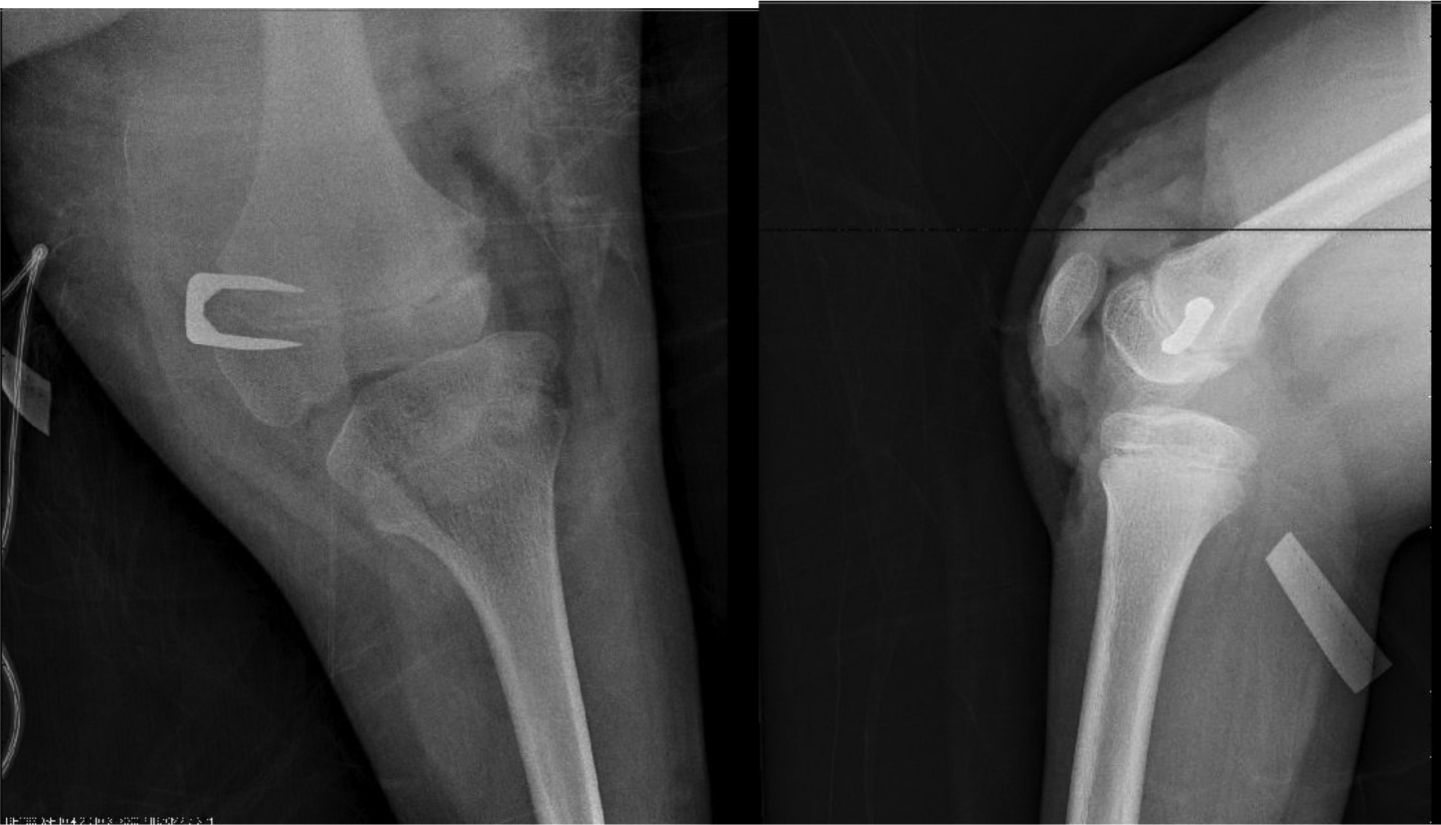
Anteroposterior and lateral radiographs of the left knee. Reduction of patellar luxation, using the modified Roux-Goldthwait technique, with medial temporary hemiepiphysiodesis to treat valgus.

**Figure 3:**
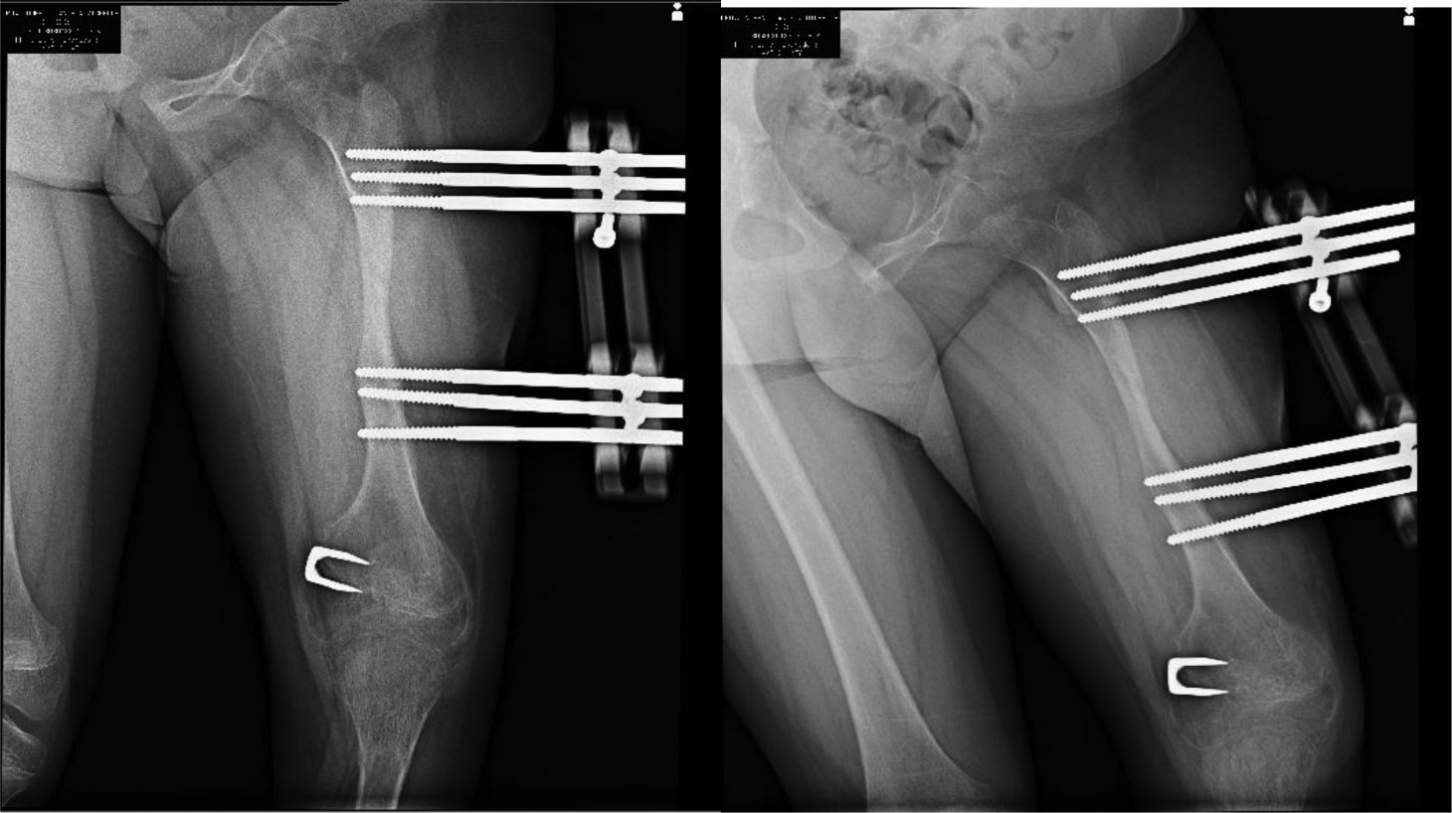
Six months postoperative anteroposterior and lateral radiographs of the left femur. Femur lengthening employing uniplanar straight-line external fixation and lengthening of 6.7 cm femoral length.

**Figure 4:**
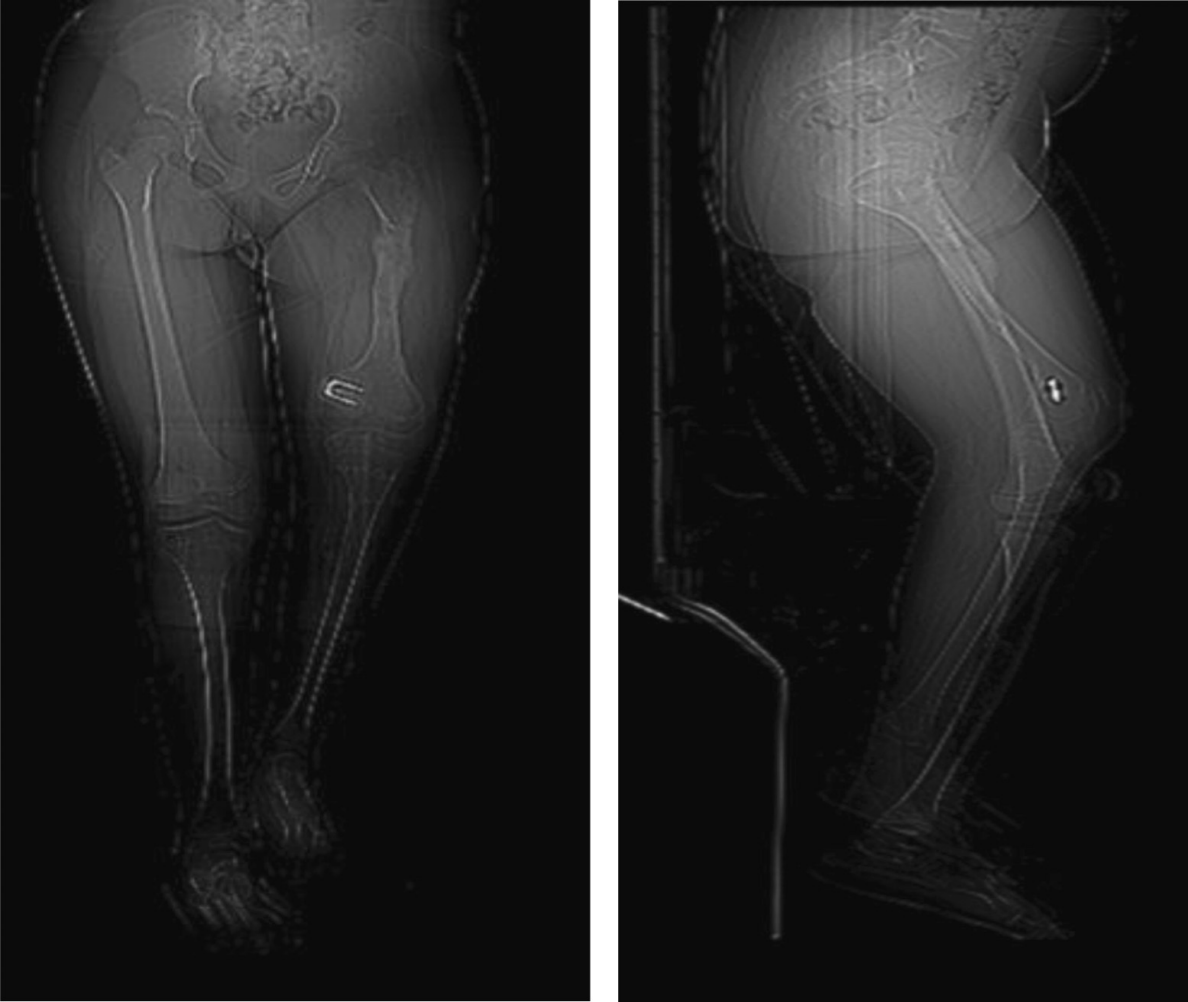
Computerized axial tomography of the lower extremities. The loss of the gain in length and the fracture of the newly formed callus are observed in the pathways of bone and knobby consolidation.

To seek definitive treatment, patellar luxation was reduced using the modified Roux-Goldthwait technique,^[8]^ using epidural anesthesia and sedation, without a tourniquet, with the patient in a supine position. A midline knee incision was made, extending 5 cm proximal to the quadriceps and 5 cm below the anterior tibial tuberosity, plane dissection was performed exposing the patellar tendon, as well as both patellar retinacula, adhesions were released, and the lateral retinaculum was resected up to 5 cm proximal to the upper pole of the patella. The patellar tendon was divided into 2 parts, and the lateral half of the patellar tendon was disinserted as distally as possible from the tibial tubercle and slid under the portion that was attached to the patella and then attached to the medial fibrous plane. Subsequently, using a Krackow suture in the tendon, subperiosteal transosseous sutures were performed using 2–0 absorbable suture (Vicryl). Likewise, plication was performed and tension was applied to the medial retinaculum with a continuous 2–0 absorbable suture (Vicryl). To treat flexion contracture, the posterior capsule was released to obtain full extension. It was not necessary to lengthen the quadriceps or goosefoot as it did not show any shortening. In the end, the position of patellar reduction was evaluated in 30° flexion, as well as in extension, and to treat the valgus deviation, utilizing medial hemiepiphysiodesis, a staple was placed with the Blount technique (Fig. 2). It was sutured by planes, and then a 1/4 hemovac and a plaster cast were placed at 10° of flexion. At 6 weeks, the cast was removed, the wound healed without complications, and the patient was referred for rehabilitation, where the muscles were strengthened, allowing active flexion and extension at 3 weeks. One year after surgery, the patella remained reduced, and the flexion contracture was −15°. Subsequently, a second surgical intervention was performed on October 26, 2017, when the patient was 9 years and 6 months old, to lengthen the femur, employing a uniplanar strip-type external fixation (Fig. 3). The dynamization of the femur elongator was performed on May 22, 2018. On November 9, 2018, after 12 months of using the bar for elongation, with a total gain of 6.7 cm in femoral length, the external fixator was removed. On December 18, 2018, during a follow-up visit, displacement in retrovarus was observed secondary to an elongation site fracture. A loss of 4.7 cm in fracture of bone elongation was presented as a complication (Fig. 4), which was treated with plaster. The patella, when clinically evaluated, remained aligned and reduced, and a 5-cm limb discrepancy persisted. The patient lost her medical service and follow-up was no longer possible.

## 4 Discussion

Clinical and radiographic abnormalities in patients with PHLE present a wide spectrum, but their combination with CDP is a rare condition. Patient age and deformities represent a challenge for treatment decisions. For CDP, surgical intervention is essential to restore patellar function and avoid a delay in the development of gait, but it must be performed before 1 year of life,^[9]^ and in the case of FH, the degree of foot deformity is decisive in assessing the need for amputation or attempt to reconstruct the limb. The decision between reconstruction or amputation in FH is complex and requires knowledge and clinical experience, which generates controversy in the literature.^[3]^ For example, our case presented 3 rays, which indicated the possibility of 49% salvage and reconstruction surgery.^[10]^ In contrast, the case also presented an absence of the fibula and PFFD in association, which meant that severe deformities that would need multiple reconstruction surgeries and use of an external fixator for prolonged periods can lead to fractures, residual deformity, and infection with a high risk of amputation. Furthermore, the number of congenital abnormalities, although it is not a prognostic predictor and does not correlate with the results of lengthening, implies prolonged treatment with various complications, because treatment begins at an old age. This should be considered when deciding on reconstruction because amputation offers a single surgical event with minimal complications.^[11]^ There is an ongoing debate regarding what factors are decisive in saving the limb, either the age at initiation of treatment or the severity of the deformities.^[12]^

In reference to this discrepancy, it was reported that asymmetry in length or deformity in the foot does not correlate with the amount of fibula absent, and that predicting skeletal maturity asymmetry is useful to determine the number of lengthening to perform. It is recommended to start tibial lengthening at 4 years, with what could be achieved up to >20 cm of tibial elongation, but femoral lengthening does not issue a recommendation. Likewise, it has been proposed to classify ankle deformities for their reconstruction in stages.^[13]^ In our case, a discrepancy of 12.6 cm was projected and, as it was accompanied by the presence of 3 rays (Paley type 1 HF), thus, surgical reconstruction was preferred. Similarly, when deciding on reconstruction, it is recommended to perform ankle reconstruction before elongation at an early age and that the fibular remnant in Achterman and Kalamchi type II deformity^[6]^ should be excised and used in the reconstruction of the lateral malleolus by functioning as a support on the ankle, which prevents not only the valgus deviation of the ankle but also of the knee; therefore, its resection avoids the anchor effect in the ankle and knee, which improves the axial mechanical axis.^[14]^ The ankle was stable in this case, and no remaining fibula was present; therefore, surgical reconstruction was preferred. However, guidelines are uncertain when CDP presents with valgus deformity as well as an absence of ligaments in the knee with TC, which must be considered for the function of the entire limb. In such cases, where elongation is performed, there is a recurrence of axial deformity in both FH and PFFD, since recurrent valgus is common in both. It has been reported that the age at initiation of treatment is not predictive of the presence of a residual axial deformity, but if it is a more severe deformity, it is likely that recurrence will occur, especially with an absent spherical knee joint of cruciate ligaments and lateral hypoplasia of the distal epiphysis of the femur.^[15]^ In the case presented here, valgus was treated with guided growth, and questions arose as to whether treating patients with guided growth, who will be subjected to bone lengthening, could decrease recurrence in axial alignment of the knee.

Finally, the decision to begin with the knee deformity was based on anatomical alignment of the knee and patellar luxation, since complications were thought to arise in limb soft tissues. Because of the planned bone elongation in the second stage, it would make it difficult to correct the limb alignment, which was achieved by medial temporary hemiepiphysiodesis.

Aspects that must be considered are pain, function, and quality of life due to the assertion that subjects treated at a younger age and in a multidisciplinary way could have similar results regarding knee function. In terms of the ankle, function is impaired at any age, and this should be considered when opting for reconstructive treatment or amputation.^[16]^

## 5 Conclusion

PHLE with FH and PFFD and associated with CDP has not been described extensively in the literature; therefore, there is no established treatment. In this case, the clinical results of valgus knee were encouraging, and guided growth could be an option to avoid valgus deformity of the knee and improve the axial deformity. Mono-axial, mono-lateral external fixator femoral lengthening may be safe under close monitoring but may be complicated by fractures with loss of gained bone length. PHLE is usually accompanied by severe deformities in the lower limb, from the knee to the forefoot; therefore, starting reconstruction at an early age together with the adequate classification of deformity are essential factors when opting for limb amputation or reconstruction.

### 5.1 Uncited reference

^[11]^.

## Author contributions

**Conceptualization**: Sergio Charles-Lozoya.

**Investigation**: Sergio Charles-Lozoya, Gibran Ruíz-Zenteno, María Lizcano-Martínez.

**Methodology**: Héctor Cobos-Aguilar, Edgar Manilla-Muñoz.

**Resources**: Adrián García-Hernández.

**Writing – original draft**: Gibran Ruíz-Zenteno, Sergio Charles-Lozoya.

**Writing – review & editing**: Sergio Charles-Lozoya, Miguel Leonardo De La Parra-

Márquez, Héctor Cobos-Aguilar.
